# DNA-Dependent Protein Kinase Inhibits AID-Induced Antibody Gene Conversion

**DOI:** 10.1371/journal.pbio.0050080

**Published:** 2007-03-13

**Authors:** Adam J. L Cook, Joanna M Raftery, K. K. Edwin Lau, Andrew Jessup, Reuben S Harris, Shunichi Takeda, Christopher J Jolly

**Affiliations:** 1 Centenary Institute and University of Sydney Faculty of Medicine, Sydney, New South Wales, Australia; 2 Department of Biochemistry, Molecular Biology, and Biophysics, University of Minnesota, Minneapolis, Minnesota, United States of America; 3 Institute for Molecular Virology, University of Minnesota, Minneapolis, Minnesota, United States of America; 4 Department of Radiation Genetics, Kyoto University School of Medicine, Kyoto, Japan; Scripps Research Institute, United States of America

## Abstract

Affinity maturation and class switching of antibodies requires activation-induced cytidine deaminase (AID)-dependent hypermutation of Ig V(D)J rearrangements and Ig S regions, respectively, in activated B cells. AID deaminates deoxycytidine bases in Ig genes, converting them into deoxyuridines. In V(D)J regions, subsequent excision of the deaminated bases by uracil-DNA glycosylase, or by mismatch repair, leads to further point mutation or gene conversion, depending on the species. In Ig S regions, nicking at the abasic sites produced by AID and uracil-DNA glycosylases results in staggered double-strand breaks, whose repair by nonhomologous end joining mediates Ig class switching. We have tested whether nonhomologous end joining also plays a role in V(D)J hypermutation using chicken DT40 cells deficient for Ku70 or the DNA-dependent protein kinase catalytic subunit (DNA-PKcs). Inactivation of the *Ku70* or *DNA-PKcs* genes in DT40 cells elevated the rate of AID-induced gene conversion as much as 5-fold. Furthermore, DNA-PKcs-deficiency appeared to reduce point mutation. The data provide strong evidence that double-strand DNA ends capable of recruiting the DNA-dependent protein kinase complex are important intermediates in Ig V gene conversion.

## Author Summary

To generate highly specific antibodies in response to an immune challenge, the antibody genes in activated B cells mutate at a very high rate over a period of several days. The enzyme that initiates antibody gene mutation is activation-induced cytidine deaminase (AID), the first protein recognized to directly edit DNA genomes in vivo. AID induces point mutation of antibody V genes in all vertebrates, as well as transfer of short sequences from nonfunctional donor V genes to functional acceptor V genes (“gene conversion”) in birds and some other species. Whether or not the mechanism of AID-induced V gene mutation and gene conversion involves double-strand DNA breaks is controversial and potentially important because double-strand DNA breaks are known to promote cancer-associated gene translocations. We used genetic inactivation of a double-strand break repair protein (DNA-dependent protein kinase) in a chicken B cell line to indirectly test whether AID induces double-strand breaks in the antibody V genes. We conclude that physiological expression of AID causes the formation of double-strand DNA ends in antibody V genes, which appear to be prevented from participating in homologous recombination if they recruit DNA-dependent protein kinase.

## Abstract 3

Affinity maturation and class switching of antibodies requires AID-dependent hypermutation of Ig V(D)J rearrangements. This process is shown here to involve double-strand DNA breaks that recruit repair machinery.

## Introduction

In humans and mice, primary antibody (Ig) diversity is produced by V(D)J recombination, which is dependent on the RAG-1 and −2 proteins [1]. Over a lifetime, primary repertoires are largely re-shaped by the processes of Ig somatic hypermutation (SHM) and class switching [2], independent processes which occur in B cells activated by infection or immunization. SHM and class switching absolutely depend on a mutator protein, activation-induced cytidine deaminase (AID or AICD), whose expression is restricted to activated B cells [3,4]. In humans and mice, Ig SHM predominantly involves point mutation of rearranged Variable (V) gene segments and the immediately downstream intron sequences, leaving the Constant region (C) gene segments largely unaffected [5,6]. In some species, including chickens, SHM of rearranged V genes also involves intra-chromosomal gene conversion with related pseudo- (Ψ) V genes, in preference to point mutation [7]. A minority (5%–10%) of AID-induced mutations in Ig V(D)J genes in all species are small deletions and insertions, which might be due to nonhomologous DNA end joining (NHEJ) and template slippage during translesion synthesis [8–11]. Although class switching also involves AID-induced point mutation, now targeted to the Switch (S) regions located upstream of each C region gene in the IgH locus [6,12–14], its salient outcome is recombination between S regions via NHEJ and the concomitant deletion of kilobase regions of DNA [1].

There is now compelling evidence that AID represents a previously unrecognized class of DNA-editing enzymes vital for both antibody diversification and direct destruction of viral DNA [15]. AID deaminates deoxycytidine (dC) bases in targeted Ig gene regions, converting the targeted bases to deoxyuridine (dU), and thus directly causes transition mutations of dC/dG (deoxycytidine/deoxyguanosine) base pairs to dA/dT (deoxyadenosine/deoxythymidine) base pairs [10]. Excision of AID-deaminated bases by uracil-DNA glycosylase (UNG) or by mismatch repair leads to further mutation via translesion DNA repair [10,16–23]. In chicken Ig V genes, excision of AID-induced dU bases by UNG mostly leads to homology-directed gene conversion with ΨV genes by a process independent of translesion DNA repair, rather than to point mutation [21,24,25]. In yeast and vertebrate cell models, gene conversion is stimulated by the induction of a double-strand break (DSB), which produces the requisite free 3′-ends [26,27]. However, this does not imply that DSBs are obligatory for gene conversion because free 3′-ends are also generated during DNA replication. It is clear that the combined attack of Ig S regions by AID and UNG results in DSBs, which are required for class switching [28,29], but there is no a priori reason to expect a role for DSBs in AID/UNG-induced point mutation or gene conversion. On the contrary, nicking at AID/UNG-induced abasic sites could even prevent mutation, promoting faithful Ig V gene conversion with sister chromatids (in S-phase) or faithful base excision repair (in G1-phase) instead [11,30]. Attempts to directly demonstrate AID-dependent DSBs in mutating Ig V genes by ligation-mediated PCR have produced mixed results [31–35]. This is probably because DNA extracted from mutating cells carries a high background of breaks caused by, for instance, normal DNA replication, apoptosis, and even mechanical damage during DNA extraction. Although the frequency of staggered double-strand DNA (dsDNA) ends detected in the VDJ_H_-rearrangement of human CL-01 cells is increased by AID overexpression, there is no convincing evidence that physiological expression of AID causes Ig V region DSBs [35].

Since NHEJ plays a role in repair of DSBs in all phases of the cell cycle [36], we tested whether NHEJ influenced Ig V hypermutation in DT40 B cells. The V(D)J-rearranged heavy- and light-chain genes in chicken DT40 cells mutate constitutively by both dC/dG point mutation and gene conversion, in an AID- and UNG-dependent manner, although the mismatch-repair-mediated dA/dT mutation pathway is essentially inactive in these cells [37,38]. We show that reducing the efficiency of NHEJ in DT40 cells by inactivating either the *Ku70* or *DNA-PKcs* genes increases the rate of gene conversion with ΨV genes, implicating DNA breaks in the mechanism of AID-induced gene conversion.

## Results/Discussion

### Loss of DNA-Dependent Protein Kinase Subunits Increases sIg Gain and Gene Conversion in DT40 Cells

Ku70, Ku86, and the DNA-dependent protein kinase catalytic subunit (DNA-PKcs) form the heterotrimeric protein DNA-dependent protein kinase (DNA-PK), which is primarily responsible for processing dsDNA ends in G1-phase vertebrate cells, protecting them from inappropriate homologous recombination, and promoting rapid, usually faithful end re-joining by DNA ligase IV [1]. Deficiency for Ku70 or DNA-PKcs was previously reported to have no effect on sIg loss in DT40 cells [39], but the possibility that Ig V gene conversion might involve DSBs prompted us to re-assess whether sIg gain was affected by NHEJ. In DT40 cells, sIg fluctuation is complicated by the fact that the Ig V gene rearrangements mutate by both point mutation and gene conversion. The donor ΨV genes have varying homology to the acceptor V(D)J genes, but in the Igλ locus (and probably also the IgH locus) they usually code for nearly complete reading frames with only a few ΨV_λ_ genes carrying premature stop codons [7]. Ig V gene conversion tracts frequently cover many codons [7]. Gene conversion is therefore a more efficient way to repair premature stop codons than is single base point mutation, because any gene conversions initiated near a deleterious mutation are biased toward repairing it. This is particularly true in the DT40-CL18 subline where sIg loss in the founder cell was due to a single base frame shift in the VJλ gene [40]. We can therefore infer that the rate of sIg gain in lines derived from CL18 cells is essentially an indirect measure of the Ig V gene conversion rate.

The DNA-PKcs- and Ku70-knockouts were originally generated in DT40 cells carrying the canonical CL18 VJλ frame shift [41], which we confirmed by DNA sequencing (unpublished data). We found that deficiency for either Ku70 or DNA-PKcs increased sIg gain relative to control CL18 cells at the 0.001 significance level (Figure 1). To our surprise, DNA-PKcs-deficiency had more of an effect on sIg gain than Ku70-deficiency: a repeat experiment where clones were cultured for 24 d, rather than 50 d, confirmed the reproducibility of these results (Figure 1B). This demonstrated that the power of sIg fluctuation analyses to detect small differences in Ig V mutation rates depends on the use of a large number of clones, rather than on the duration allowed for mutations to accumulate—a conclusion consistent with mathematical modeling of DT40 sIg fluctuation [42].

**Figure 1:**
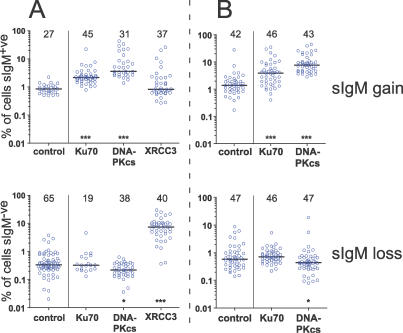
Surface Ig Reversion of DT40 Clones Accumulation (top) of sIg^+ve^ cells in sIg^−ve^ DT40 clones, or (bottom) of sIg^−ve^ cells in sIg^+ve^ DT40 clones after (A) 50 d of culture, or (B) 24 d of culture in two independent experiments (A or B) is depicted. The protein missing from each cell line is indicated. Control cells were (top) sIg^−ve^ DT40-CL18 cells, or (bottom) sIg^+ve^ DT40-CL18 cells. Each circle represents the frequency of sIg-reverted cells (on a log scale) detected in each clone by FACS after the culture period. The bar indicates the median reversion frequency. The numbers above each dataset give the number of clones analyzed. Asterisks indicate significant difference of the median reversion frequency from the control population: * *p* < 0.05, *** *p* < 0.001. Note: lack of surface Ig-expression in the *XRCC3^−/−^* founder clones was due to Ig V(D)J point mutations and not due to the canonical CL18 frame shift carried by the control, *Ku70^−/−^*, and *DNA-PKcs^−/−/−^* clones (unpublished data). Thus, the reduced rate of Ig V gene conversion known to exist in these cells [43,45] did not significantly reduce their rate of surface Ig gain.

Similar phenotypes in two independent knockouts acting in the same DNA repair pathway (NHEJ) made it unlikely that the observed increases in sIg gain were artifacts due to unknown additional mutations. Nor were the increases due to preferential outgrowth of sIg^+ve^ cells in the NHEJ-deficient cultures, because the cloning efficiency of sIg^−ve^ and sIg^+ve^ NHEJ-deficient cells was the same (unpublished data). In our cultures, the doubling times for CL18, *DNA-PKcs^−/−/−^*, and *Ku70^−/−^*DT40 cells in log-phase growth were 11.2 h, 11.6 h, and 12.0 h, respectively. Using these doubling times in mathematical modeling [42] of our fluctuation data suggested that the mean rate of sIg gain in DNA-PKcs- and Ku70-deficient cultures was 5.0× and 2.9× that of control cells, respectively (Table 1).

**Table 1:**
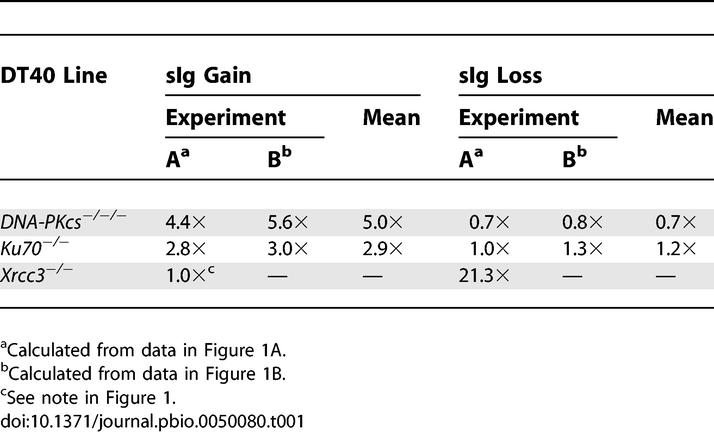
Estimated Changes in sIg Reversion Rates Relative to CL18 Control Cells

VJ_λ_ rearrangements PCR-amplified from random Ku70- and DNA-PKcs-deficient DT40 clones carried more Ig V_λ_ gene conversions than those derived from control CL18 clones grown in parallel, while no gene conversions were detected in DNA amplified from control *AID^−/−^*cells (Figure 2 and Table 2). The relative increases in Ig V gene conversion detected by sequencing were not large (Table 2), but this was probably due to sampling error. The effective sampling rate of the sIg fluctuation assay is much higher than that of DNA sequencing because the mutagenic gene conversion rate of DT40 cells is fairly low. We chose not to overexpress AID as a way of counteracting this problem because variation in AID overexpression could have greatly increased data variance, and because mutation by overexpressed AID may not reflect physiological AID-induced mutation. The sequence data were consistent with the statistically highly significant increases in the rates of sIg gain calculated in Table 1. Furthermore, sequencing of 93 VJλ-rearrangements from CL18, *DNA-PKcs^−/−/−^*, and *Ku70^−/−^* clones, which started from sIg^+ve^ cells (part of the dataset shown in Table 2), confirmed that sIg gain in these cells always involved a gene conversion that removed the canonical VJλ frame shift (unpublished data). Overall, the sequence data confirmed that both DNA-PKcs- or Ku70-deficiency increased Ig V gene conversion.

**Figure 2:**
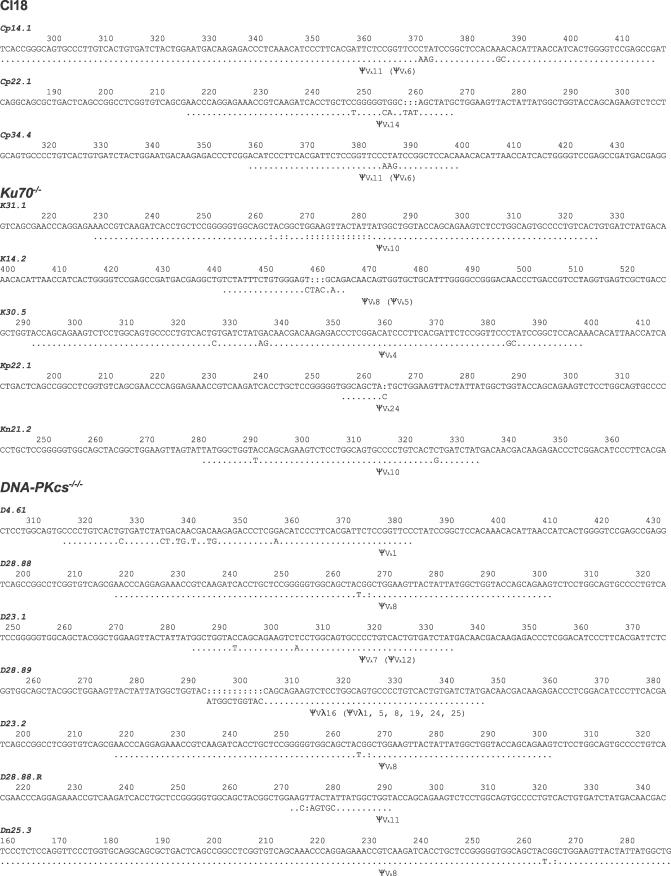
Gene Conversions Detected in CL18, *DNA-PKcs^−/−/−^*, and *Ku70^−/−^* Cell Lines The consensus sequence for each clone is shown at the top and the mutated sequence carrying a gene conversion underneath. Dots indicate identity to the clone's starting (consensus) sequence and indicate the maximum extent of sequence that could have undergone gene conversion. The ΨV_λ_ gene with the longest stretch of identity to the mutated sequence is indicated as the gene conversion donor, with other possible donors listed in parentheses. (The identity in clone Cp14.1 with the “best” donor ΨV gene extends 5′ to that shown in the figure. Similarly, identity in clone Dp25.3 extends 3′ to that shown in the figure.) Colons indicate the insertion of a gap to maintain sequence alignment. Numbering indicates the sequence position of each gene conversion, with the translation start as base 1. Note: The same gene conversion with ΨV_λ_ occurred independently in two *DNA-PKcs^−/−/−^*clones.

**Table 2:**
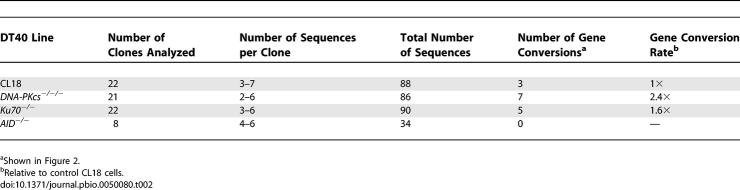
Gene Conversions Detected by Sequencing Random Clones from Experiment A

### DNA-PKcs-Deficiency Reduces sIg Loss and Ig V Point Mutation

In addition to the obvious increase in sIg gain, we were able to measure a small and reproducible decrease in sIg loss in DNA-PKcs-deficient cells, but not Ku70-deficient cells (Figure 1 and Table 1). Point mutation is far more likely to produce deleterious amino acid changes than it is to repair them. Thus, sIg loss should be more sensitive than sIg gain to changes in point mutation rates. This is illustrated by cells deficient for any of the Rad51-paralogs [39], such as *XRCC3* ^−/−^ cells, which were included in one of our sIg fluctuation assays as a control (Figure 1A). XRCC3-deficiency caused little change in the rate of sIg gain in DT40 cells but had a dramatic effect on the rate of sIg loss (Figure 1A). Thus, the inverse changes in sIg gain and sIg loss in DNA-PKcs-deficient cells (Figure 1; Table 1) suggested that loss of the DNA-PKcs protein both promoted gene conversion and inhibited point mutation in DT40 cells. The data from *XRCC3^−/−^* cells also demonstrated the ability of our mathematical modeling [42] to estimate changes in mutation rate. The 21× increase in the rate of sIg loss estimated for *XRCC3^−/−^*cells (Table 1) corresponds well to their rate of point mutation determined by sequencing [43].

Reliable point mutation data were not collected in our initial sequencing of VJλ sequences because point mutations occurred in a control *AID^−/−^* dataset (unpublished data) and were therefore largely due to errors introduced by the “BioXact” polymerase mix used for PCR. However, sequencing of an additional 40–47 clones amplified with “Phusion” DNA polymerase (Finnzyme) yielded five, three, and zero point mutations in VJλ genes amplified from CL18, *Ku70^−/−^*, and *DNA-PKcs^−/−/−^* cells, respectively (Table 3). Combined with the data summarized in Table 1, the sequence data indicate that increased gene conversion in DNA-PKcs-deficient DT40 cells is accompanied by reduced point mutation. Intriguingly, point mutation was either not reduced by Ku70-deficiency or the reduction was too small to be measured in our experiments.

**Table 3:**
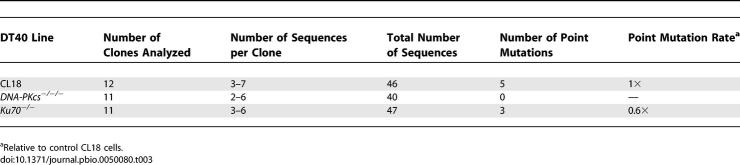
Point Mutations Detected by Sequencing Random Clones from Experiment A

### Are AID-Induced DSBs Involved in Ig V Gene Mutation?

Increased Ig V gene conversion in DNA-PK-deficient DT40 cells implies competition between DNA-PK and homology-directed repair (HDR) factors for access to hypermutating Ig V genes in wild-type DT40 cells. How might DNA-PK be recruited to mutating Ig V genes? The generation of DSBs in Ig V genes is the most obvious mechanism, although we cannot rule out the possibility that DNA-PK or its subunits play roles in Ig V mutation independent of NHEJ. AID could generate staggered Ig V DSBs in any phase of the cell cycle if two AID-bearing complexes attacked both strands of a V gene and thus recruited UNG and an abasic site-endonuclease or -lyase activity (Figure 3A). However, this scenario is unlikely to be a major cause of DSBs in DT40 cells because the rate of attack of the DT40 VJλ gene by AID (as revealed in UNG- and ΨV-deficient DT40 cells [25,38,44]) is not high enough for AID-induced cleavage of both strands of an Ig V gene to occur very frequently. Alternatively, dsDNA ends (“pseudo”-DSBs) could also be produced during S-phase if a replication fork encountered a single-strand AID-induced nick (Figure 3B). In both Figure 3A and 3B, resection of dsDNA ends would produce 3′-extensions capable of initiating gene conversion with ΨV genes; dsDNA ends with both 5′- and 3′-extensions have been detected by LM-PCR in rearranged Ig V genes in human B cells expressing AID [35]. Our data provide indirect, less artifact-prone confirmation that physiological expression of AID does indeed generate dsDNA ends in Ig V genes.

**Figure 3:**
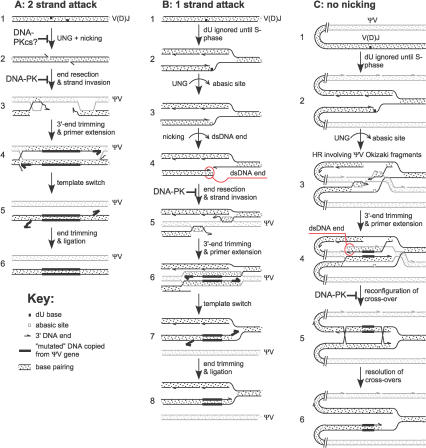
Models for Ig V Gene Conversion Based on Attack of Both DNA Strands by AID and Attack of One DNA Strand by AID (A) Attack of both DNA strands by AID. In step 1, two complexes containing AID attack opposed strands at the same time. If both complexes directly recruited DNA-PKcs molecules, DNA-PKcs could dimerize and perhaps inhibit base excision by UNG. In step 2, excision by UNG and a lyase or exonuclease creates a staggered DSB. Step 3 shows that gene conversion requires the production of 3′-protruding ends, which may involve a 5′–3′ exonuclease, depending on the relative placement of the nicks. The 3′-protruding ends initiate gene conversion by invading a homologous ΨV gene. This step could be inhibited by the binding of dsDNA ends to the DNA-PK complex. In steps 4–6, nonhomologous (“mutated”) sequences are copied from the ΨV gene by mismatched end trimming, primer extension, template switch, further end trimming, and ligation. (B) Attack of one DNA strand by AID. In step 1, AID deamination in G1-phase may be ignored until S-phase, based on the model in [11]. In S-phase, shown in step 2, the dU base produced by AID is encountered by a replication fork, enabling access by UNG, which then creates an abasic site. Step 3 shows the abasic site is excised to create a dsDNA end, which can recruit the DNA-PK complex. In step 4, if DNA-PK is not recruited, the dsDNA end promotes gene conversion with an upstream ΨV gene. (C) Attack of one DNA strand by AID. As in (B), AID deamination is ignored until S-phase. In step 2, as in step 2 of (B), the dU base is encountered by a replication fork, but in this case lagging strand nicks (Okazaki fragments) are still present in the upstream ΨV genes. Step 3 shows the abasic site generated by UNG stalls the replication fork, without inducing nicking, and promotes strand invasion into an upstream ΨV gene. Step 4 shows a dsDNA end is created when primer extension encounters a lagging strand nick due to Okazaki fragments in the template ΨV gene. The replication fork stalls again. If DNA-PK binds the dsDNA end it inhibits the completion of HDR. In step 5, a second reconfiguration of the stalled replication fork occurs, and in step 6, resolution of the double cross-over completes gene conversion.

Di Noia et al. [11] have recently argued that AID-induced dC/dG point mutation need not involve DNA breaks. Indeed, in Rad51 paralog-deficient cells, AID/UNG-induced abasic sites must be diverted from gene conversion to point mutation prior to excision of the abasic site, otherwise no lesion would be present to recruit translesion bypass and transversion point mutation [19,39,45]. This is illustrated in Figure 3A and 3B, where only intermediates 3 and 4 can divert to translesion bypass. We were able to envisage a scenario where NHEJ could inhibit Ig V conversion independently of nicking at abasic sites, but the scenario required nicks between Okazaki fragments to persist in template ΨV genes for some time after the replication fork had passed (Figure 3C). This requirement is more likely to be met in chicken B cells than in human or mouse B cells (which do not undergo AID-induced gene conversion) because the V genes are much closer together in chicken B cells, and furthermore, is consistent with the preferential use of closer ΨV genes as gene conversion donors [7]. However, we think scenario B in Figure 3 is more likely than scenario C, because it is clear that the combined activity of AID and UNG recruits DNA nicking to Ig S regions participating in switching [28]. Thus, it is reasonable to expect the same in Ig V regions. Nonetheless, there is no data available to rule out scenario C in Figure 3 yet.

We conclude that inhibition of mutagenic gene conversion by DNA-PK strongly implicates dsDNA ends as frequent, even obligatory precursors of Ig V gene conversion. The production of a dsDNA end by any of the scenarios shown in Figure 3 provides two 3′ DNA ends that can simultaneously prime strand invasion into an upstream ΨV gene. Trimming of mismatched 3′-ends (which frequently occurs when nonidentical sequences participate in HDR [46]) after simultaneous strand-invasion provides a simple mechanism by which both strands of the ΨV gene are copied into the acceptor V(D)J gene (Figure 3). A good candidate for the nicking enzyme required for models A or B in Figure 3 is the abasic site-lyase activity of MRE11/RAD50 [47].

In contrast to inactivation of DNA-PK, the inactivation of Rad51 paralogs causes a dramatic increase in point mutation in DT40 cells [39]. Thus, the ability of NHEJ to compete with Ig V gene conversion does not, at first glance, appear to be comparable to the ability of HDR to inhibit translesion bypass. However, this is probably because the majority of Ig V gene conversions induced by AID are non-mutagenic: using ΨV genes, which have regions of identity to the 3′ acceptor VJ gene or the sister chromatid, as repair templates. Thus, any increase in Ig V gene conversion increases the rate of faithful gene conversion as much as it increases mutagenic gene conversion. In fact, it is only when gene conversion is inhibited that the rate of attack of Ig V genes in DT40 cells by AID is “unmasked” as being much higher than the mutation rate of wild-type DT40 cells would suggest [25,39,44,45]. A 2- to 5-fold increase in mutagenic gene conversion in the absence of DNA-PK therefore implies that DNA-PK in fact blocks Ig Vλ gene conversion most of the time.

### A Ku-Independent Role for DNA-PKcs in Ig V Gene Mutation?

Gene conversion-mediated repair of I-Sce I-induced DSBs is elevated much more by Ku70-deficiency than it is by DNA-PKcs-deficiency [41], probably because Ku70 directly competes with the gene conversion machinery for access to dsDNA ends, while DNA-PKcs does not. This contrasts with AID-induced Ig V gene conversion, where DNA-PKcs appears to be more inhibitory than Ku70 (Tables 1 and 2). Perhaps some of the inhibitory activity of DNA-PKcs is independent of Ku70. Wu et al. showed that DNA-PKcs, and not Ku, associates with AID in a DNA-dependent manner [48]. It is unclear whether the reported association between AID and DNA-PKcs was physiological because it was enhanced by addition of exogenous DNA [48]. Nonetheless, one can speculate that in wild-type cells simultaneous recruitment of AID to both DNA strands of a V gene could directly promote DNA-PKcs dimerization, partially inhibiting access by UNG or HDR factors to the deaminated site, and thus inhibiting gene conversion whilst promoting point mutation (Figure 3A).

### Relevance to Mammalian B Cells

The *scid* mutation, which is generally considered to essentially inactivate DNA-PKcs, has no detectable effect on Ig V hypermutation in mouse Peyer's patch B cells [49]. However, this finding needs to be interpreted cautiously. Ig class switching is only reduced 50%–60% by the mouse *scid* mutation [13], in contrast to the almost complete abrogation of switching in mouse *DNA-PKcs^−/−^* cells [50], proving that the *scid* form of DNA-PKcs is surprisingly functional in some NHEJ reactions. With the exception of gene conversion, the mechanism of AID-induced mutation so far appears to be very similar in mouse, human, and chicken B cells, so it is reasonable to predict that the DNA-PK complex is recruited to mutating Ig V genes by physiological AID-activity in human and mouse Ig V genes. Our data support the possibility that a subset of human oncogenic translocations that involve Ig V genes are a consequence of hypermutation, rather than V(D)J-recombination, occurring via a DSB-induced mechanism similar to “switch” translocation [9].

## Materials and Methods

### Antibodies and reagents.

All chemicals were supplied by Sigma-Aldrich (http://www.sigmaaldrich.com), unless otherwise stated. Complete culture medium was RPMI, supplemented with 10% fetal bovine serum (lot 092K2300), 1% chicken serum (IMVS, http://www.imvs.sa.gov.au), benzyl penicillin (0.06 g/l), and streptomycin sulphate (0.1 g/l).

### Cell culture.

The DT40 subline CL18 and *AID^−/−^*, *Ku70^−/−^*, *DNA-PKcs^−/−/−^*, and *XRCC3^−/−^* DT40 lines produced by gene-targeting have been described before [40,41,51,52]. Aliquots (∼10^6^ cells) of DT40 cells were stained with saturating amounts of FITC-conjugated anti-chicken IgM Ab (clone M-1, Southern Biotechnology Associates, http://southernbiotech.com) at 4 °C for 30 min in sterile PBS containing 0.5% (w/v) BSA and 0.02% (w/v) sodium azide (PBA). Using a FACSVantage DIVA sorter (Becton Dickinson, http://www.bd.com), sIg^+ve^ or sIg^−ve^ cells were pre-sorted to enrich the rarer population and allowed to expand in culture for several days. Direct sequencing of VJ_λ_ PCR products amplified from sIg^−ve^ *DNA-PKcs^−/−/−^* and *Ku70^−/−^*DT40 cells after pre-sorting confirmed that the overwhelming majority of sIg^−ve^ cells in these lines carried the canonical CL18 frame shift (unpublished data). PCR amplification across the *DNA-PKcs* and *Ku70* exons deleted by gene targeting was also performed to confirm correct genotypes for the cells recovered from pre-sorting (unpublished data). Following expansion in culture, pre-sorted cells were sorted again for single sIg^+ve^ or sIg^−ve^ cells into either 384-well (experiment A) or 96-well (experiment B) tissue culture plates, containing complete culture medium plus Primocin antibiotic (InvivoGen, http://www.invivogen.com). 8 d after sorting, random clones were transferred to individual wells in 96-well (experiment A), or 24-well (experiment B) plates. Thereafter, when most clones had reached a density of 1 to 2 × 10^6^ cells per ml (generally at 2- to 3-d intervals), 1/20th of each culture was transferred to a fresh well containing 0.2 ml (experiment A) or 1.0 ml (experiment B) fresh medium until analysis on day 50 (experiment A) or day 24 (experiment B).

### Analysis of surface Ig-loss and Ig-gain (sIg fluctuation).

For FACS, ∼80% of each culture was harvested into 1-ml tubes, pelleted (500*g*, 5 min), and stained with FITC-conjugated anti-chicken IgM Ab in a 50-μl volume of ice-cold PBA. After washing with ice-cold PBA, cells were fixed with 2% paraformaldehyde in PBS. Clones of the same genotype and sIg phenotype were grown side-by-side in the multi-well plates and Ab-stained for FACS-analysis in tubes arrayed in a similar format. This ensured that any cross-contamination that might occur between wells during culture or Ab-staining would most likely only occur between clones of the same genotype and starting phenotype and would thus cause minimal distortion of the results. After fixing the Ab-staining, samples were randomized (http://www.random.org) prior to collection of the FACS data using a FACScan machine (Becton Dickinson). The frequency of sIgM^+^ or sIgM^−^ cells in each sample was then determined blind using FlowJo software (Tree Star Incorporated, http://www.treestar.com) and the gating strategy of Arakawa et al. [37]. Data on a median of 3.6 × 10^4^ viable cells (defined by forward- and side-light scatters) were collected for each sample. The experiments were designed to ensure that differences in sIg fluctuation rates between groups did not arise because of fluorescent antibody detachment over time, variations in machine parameters over the course of data collection, or because of human bias in data analysis.

### Mathematical modeling.

The frequencies of sIg-gain and sIg-loss were estimated using the formula shown in Appendix 2 of [42]. Let *f* be the median proportion of cells that gained sIgM in sIgM^−^ clones. Let *b* be the median proportion of cells that lost sIgM in sIgM^+^ clones. Let *g* be the number of generations in the experiment, calculated from the cell line's doubling time. Let *r* = *b/f.* Let *w* = [(ln(*g*) – ln(*f* + *b*))/ln(2)] – 1. Let *s* = 2 × (1 − *e* ^ln(1 −*b − f)/(g − w*)^). Then the estimates, Φ and β of the rates of sIg-gain and sIg-loss, respectively, are Φ = *s*/(*r* + 1) and β = *s* – Φ.

### DNA sequencing.

Genomic DNA was extracted from multiple random clones cultured for 50 d (i.e., experiment B). The VJ_λ_ exon and 3′-flanking sequences were PCR-amplified with published primers [39] using “BioXact Short” DNA polymerase mix (Bioline, http://www.bioline.com) or “Phusion” DNA polymerase (Finnzymes Oy, http://www.finnzymes.fi) and cloned into plasmids. To minimize the acquisition of redundant sequences, only a few plasmids derived from each clone were sequenced, as indicated in Tables 1–3.

## Supporting Information

### Accession Numbers

The Ensembl (http://www.ensembl.org) accession numbers for the genes discussed in this paper are *AID* (ENSBTAG00000018849), *DNA-PKcs* (ENSGALG00000012914), *Ku70* (ENSGALG00000011932), and *XRCC3* (ENSGALG00000011533).

## Abbreviations

AID: activation-induced cytidine deaminase
dA: deoxyadenosine
dC: deoxycytidine
dG: deoxyguanidine
DNA-PK: DNA-dependent protein kinase
DNA-PKcs: DNA-dependent protein kinase catalytic subunit
DSB: double-strand DNA break
dsDNA: double-stranded DNA
dT: deoxythymidine
dU: deoxyuridine
HDR: homology-directed repair
Ig: primary antibody
NHEJ: nonhomologous end joining
SHM: somatic hypermutation
UNG: uracil-DNA glycosylase
